# Research involvement among undergraduate health profession students in a resource-limited setting: awareness, attitude, motivators and barriers

**DOI:** 10.1186/s12909-022-03320-y

**Published:** 2022-04-06

**Authors:** Blaise Kiyimba, Linda Atulinda, Racheal Nalunkuma, Ignatius Asasira, Jonathan Kabunga, Davis Banturaki, Anastacia Ssebbowa Nabyonga, Rachel Nakiganda, Rachael Ndyabawe, Jonathan Nkalubo, Nelson Ssewante, Felix Bongomin, Sabrina Bakeera-Kitaka

**Affiliations:** 1 School of Medicine, College of Health Sciences, Makerere University, Kampala, Uganda; 2 Mulago National Referral Hospital, Kampala, Uganda; 3 Department of Medical Microbiology and Immunology, Faculty of Medicine, Gulu University, Gulu, Uganda; 4 Department of Pediatrics and Child Health, School of Medicine, College of Health Sciences, Makerere University, Kampala, Uganda

**Keywords:** Research, Undergraduate, Health, Students, Uganda

## Abstract

**Background:**

Involvement of undergraduate health professions students (HPS) in research will facilitate evidence-based clinical practice among future healthcare practitioners. This study aimed to assess research involvement among undergraduate HPS students and associated factors in Uganda.

**Methods:**

A cross-sectional study was conducted using an online assessment tool sent through WhatsApp groups and E-mail addresses of HPS in 12 medical schools in Uganda between 20th September and 5th October 2021.

**Results:**

We enrolled 398 participants with a mean age of 23.9 ± 3.7 years. Of this, 267 (67.1%) were male. One hundred twenty (30.2%) participants previously participated in a research activity: 90 (58.4%) as research assistants, 39 (25.3%) published as first authors, and 25 (16.2%) as co-authors. Training on the conduct of research was received by 242 (65.8%) participants, and 326 (81.9%) had intentions of conducting research in the future. Factors influencing participation in research activities were, age ≥ 25 years (adjusted odds ratio (aOR): 1.9, 95% confidence interval (95% CI): 1.2–3.2, *p* = 0.012), being male (aOR: 2.1, 95%CI: 1.2–3.6, *p* = 0.008), and being in a clinical year i.e., year 3 (aOR: 3.2, 95% CI: 1.1–9.3, *p* = 0.033), year 4 (aOR: 3.3, 95% CI: 1.1–9.5, *p* = 0.028) and year 5(aOR: 11.6, 95% CI: 3.2–42.1, *p* < 0.001). Lack of funds (79.6%), and mentorship (63.3%) were reported as major barriers to research.

**Conclusions:**

Despite a high proportion of HPS showing interest in getting involved in research, less than one-third reported previous involvement. Addressing barriers such as funding could potentially improve research involvement and output among undergraduate HPS in resource-limited settings.

## Background

Globally, medical research and innovation remains a basic cornerstone upon which new advancements and guidelines in clinical practice are based [1]. Provision of adequate health care services for the best patient management outcomes is pivoted on the interplay between health research scientists whose work is more dedicated to carrying out medical research and clinicians who are mainly in direct patient care [2]. A balance between these two professions is therefore crucial for continuous delivery of evidence-based care [3]. Unfortunately, the medical field is still and has for decades suffered a global shortage of health research scientists [3–5], a problem which if not curbed early could retard progress in evidence-based clinical practice [2].

HPS are generally looked at as the primary pool from which emerge majority of the various health care professionals with clinicians and health research scientists inclusive. However, a great tendency to later prefer clinical practice to health research as a career has been reported among many HPS worldwide [6, 7] despite many of them showing good attitudes towards scientific research earlier [8–11]. This has become among the leading causes of insufficient health research-scientists worldwide. Delayed exposure to research during undergraduate HPS’ training has been a commonly reported reason for this imbalance [12, 13]. Though the primary objective of undergraduate HPS’ education is to train students in providing safe and effective patient care [14, 15], the expeditious advancements in the health care system and the increasing amount of easily accessible information demand that health care practitioners make decisions based on reliable scientific evidence [15].

Sub-Saharan Africa (SSA) has continued to suffer the world’s biggest burden of disease and mortality. This has been linked to its suboptimal quality of health care delivery, which is fueled by insufficient research evidence [16]. Evidence can only be derived through carrying out high standard quality research to generate local data based on common health problems that can be used to inform guidance [16]. Despite employing various interventions to boost research such as incorporating research methods into education curriculum by many African countries [17, 18], the overall research output from SSA is still low [19]. This paucity of research has led to over dependence by many African countries’ clinical practice on research findings from developed countries, which have different disease burden and level of medical advances compared to SSA. This could result in undesirable outcomes as observed in West Africa where shortage of skilled clinical scientists just fueled disease progression and mortality instead of its containment during the Ebola virus disease outbreak [20].

In Uganda, despite the increasing number of undergraduate HPS’ schools in the last two decades from only two in 2003 to now 12 in 2021 [21], the volume of undergraduate research output has slightly improved, but still very low. In 2003, research done at one public HPS’ school reported that the major barriers for students to do undergraduate research were lack of collaborations, lack of guidance and lack of funding [22]. However, this study was done 18 years ago when the country had only two undergraduate HPS’ schools and involved only one HPS’ school.

Because different HPS’ schools may operate on different curricular, timetables, and administrative bodies, it is critical to know whether similar factors exist currently in other public and private universities, or they differ and in the different health care (HC) courses offered. Therefore, in this study, we aimed to assess research involvement of undergraduate students exploring awareness, barriers and motivators in all the 12 undergraduate HPS’ schools in Uganda.

## Methods

### Study design

Between 20^th^ September and 5^th^ October 2021, we conducted an online, descriptive and cross-sectional study across 12 universities in Uganda.

### Study area and setting

The study was conducted in Uganda. There are currently 54 universities in Uganda turning out over 40,000 graduates annually. However, only 12 universities offer health professional courses with an estimated population of 10,000 students. These include both private and public Undergraduate HPS’ schools and they are Makerere University (MAK), Mbarara University of Science and Technology (MUST), Busitema University (BU), Kabale University (KU), Gulu University (GU), Kampala International University (KIU), King Caesar University (KCU), Uganda Christian University (UCU), Muni University, Soroti University, Lira University, and Islamic University in Uganda (IUIU). MAK, GU, BU, MUST, Muni, Kabale and Soroti are public universities whilst the rest are private.

### Target population

All undergraduate students, 18 years or older, from year 1 to year 5 of study pursuing a health profession program at any of the above-mentioned universities. Programs included were Bachelor of Medicine and Surgery (MBChB), Bachelor of Biomedical Sciences (BSB), Bachelor of Nursing/Midwifery (BSN/MW), Bachelor of Pharmacy (BPHARM), Bachelor of Dental Surgery (BDS), Bachelor of Medical Radiography (BMR), Bachelor of Science in Anesthesia (BSA), among others. The estimated target population was 10,000 students.

### Sample size

A sample size of 420 participants was calculated using the modified Kish—Leslie formula for infinite population, with a prevalence of 50%, margin of error of 5% at 95% confidence interval, and a 10% non-response rate.

### Study variables

The independent variables included were sex, age, year of study, university of study, type of university ownership and program of study. Dependent variables included questions on students’ awareness about research, attitudes, anticipated motivational factors and barriers for research involvement.

### Data collection tool

The questionnaire used had 33 questions and was adopted from previously validated questionnaires by Sayedalamin et al. [19] and Lloh et al. [20]. It consisted of 5 sections as below:

- Section l. Had 7 Questions about participants’ demographics.
- Section II. Had 10 questions assessing for participants’ awareness about Research.
- Section III. Had 9 questions, assessing for participants’ attitudes towards research.
- Section IV. Had 3 questions, assessing for participants’perceived motivational factors and benefits for engaging in research.
- Section V. Had 4 questions, assessing for participants’ perceived barriers for research involvement and intentions of doing research as a career.

### Data collection procedure

Data was collected by convenience sampling method. The survey link to the online questionnaire was sent to eligible participants via students WhatsApp groups, personal WhatsApp inboxes plus personal email addresses. We assigned a research assistant to each class and program of study per participating HPS’ school. These were mainly influential people of good reputation among their colleagues, such as class representatives and program association leaders, who continuously shared the link to all the eligible WhatsApp groups and students in their contacts inviting students to participate in the study. To reduce bias due to imbalances/ poor representation of participants from different programs, year of study and school of study among others, we ensured that we assign data collectors in each category and in numbers appropriate to the population of students per category. The questionnaire was self-administered written in simple English for effective understanding by the participants.

### Quality assurance

The questionnaire was pre-tested among 15 undergraduate students from the College of Veterinary Medicine, Makerere University, and the identified corrections necessary were made before administering the tool to the final study participants. The questionnaire had check points that ensured that only completed forms could be submitted, and that each participant could submit only one response form, hence excluding duplication of responses from participating more than once.

### Data management and analysis

Upon completion of data collection, entries were downloaded. Data cleaning and coding were done using Microsoft Excel 2016 and coded data exported to STATA 15.0 for analysis. Demographic characteristics, awareness, barriers, benefits, and motivational factors to participate in research were first summarized as in tables with frequencies and percentages for categorical variables and mean and standard deviation for numerical variables. Attitude was summarized on a figure format. Associations between independent and dependent variables were assessed using Chi-square or Fisher’s exact tests. Multivariable logistic regression was performed adjusting for confounders (course and institutions). A *p* < 0.05 was considered statistically significant.

## Results

A total of 406 responses were obtained. After data cleaning, 398 entries were eligible for analysis (response rate, 398/420 (95%).

### Demographic characteristics of respondents

**Fig. 1:**
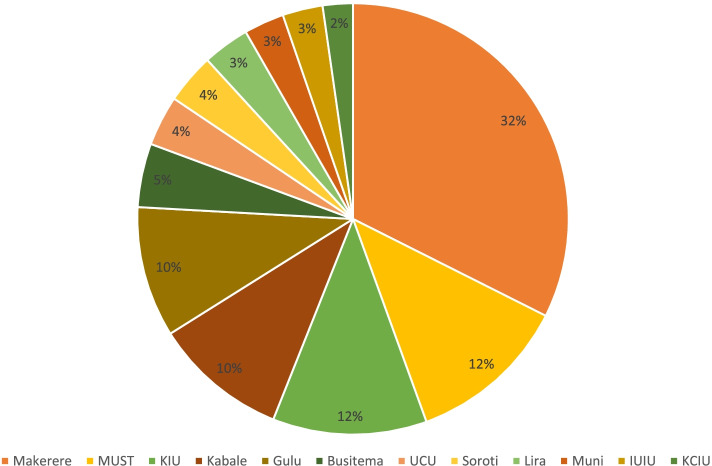
Distribution of participants across all the universities

Of the 398 respondents, 267 (67.1%) were male, 220 (55.3%) were pursuing MBChB, and 307 (77.1%) were from public universities (Fig. 1). The mean age of the respondents was 23.9 ± 3.7 years. Other demographic characteristics are presented in Table 1.

**Table 1:** Participants’ characteristics that influence participation in research activities

Variable	Total	Participation in research				
		Chi-square/Fischer’s exact test			Binary logistic regression	
	N (%)	Yes, n (%)	No, n (%)	***P***-value	aOR (95% CI)	***p***-value
Total	398 (100)	120(32.5)	249 (67.5)	N/A		
**Age**				0.001		
< 25	302 (75.9)	78 (28)	201 (72)		Reference	
≥ 25	96 (24.1)	42 (46.7)	48 (53.3)		1.9 (1.2–3.2)	0.012
***Mean (SD)***	***23.9 (3.7)***	***24.6 (3.5)***	***23.5 (3.8)***			
**Sex**				0.002		
Female	131 (32.9)	26 (21.7)	94 (78.3)		Reference	
Male	267 (67.1)	94 (37.8)	155 (62.2)		2.1 (1.2–3.6)	0.008
**Course**				0.246		
MBChB	220 (55.3)	73 (35.6)	132 (64.4)		Reference	
BDS	10 (2.5)	1 (11.1)	8 (88.9)		1.1 (0.5–2.2)	0.780
BNUR	52 (13.1)	16 (33.3)	32 (66.7)		0.2 (0–2.1)	0.183
BPARM	44 (11.1)	8 (20)	32 (80)		0.5 (0.2–1.3)	0.177
Others	72 (18.1)	22 (32.8)	45 (67.2)		1 (0.5–2)	0.964
**Year of study**				< 0.001		
Year 1	43 (10.8)	5 (13.5)	32 (86.5)		Reference	
Year 2	111 (27.9)	26 (26.3)	73 (73.7)		2.1 (0.7–6)	0.176
Year 3	97 (24.4)	32 (35.2)	59 (64.8)		3.2 (1.1–9.3)	0.033
Year 4	119 (29.9)	38 (33.3)	76 (66.7)		3.3 (1.1–9.5)	0.028
Year 5	28 (7)	19 (67.9)	9 (32.1)		11.6 (3.2–42.1)	< 0.001
**University ownership**				0.426		
Private	91 (22.9)	24 (28.9)	59 (71.1)		Reference	
Public	307 (77.1)	96 (33.6)	190 (66.4)		0.8 (0.4–1.5)	0.527
**Student’s education funding status**				0.224		
Government funded	145 (36.4)	38 (28.6)	95 (71.4)		Reference	
Private funded	253 (63.6)	82 (34.7)	154 (65.3)		1.3 (0.8–2.3)	0.282

N: Total sample, n: proportion of the sample, *N/A* Not applicable, *aOR* Adjusted odds ratio, *CI* Confidence interval

### Awareness about research

Most (92.7%, *n* = 369) respondents had ever heard of the concept of medical research and 297 (80.7%) knew a colleague who had participated in research. One-hundred and twenty (32.6%) respondents had personally participated in research outside academic requirements. Of this, 90 (58.4%) participated as research assistants, 39 (25.3%) as principal investigators and 25 (16.2%) as co-investigators. Of those that had participated in research before this survey, 27 (22.5%) had published a paper in a peer-reviewed journal. Twenty-one (70%) of the 27 publications were in international journals.

With regard to research-related training, 242(65.8%) respondents reported to have had prior training in proposal writing, 101(27.4%) manuscript writing and 68(18.5%) publication process (Table 2).

**Table 2:** Responses to awareness questions

Question	Frequency	Percent
**Have you ever heard about medical research? (*N*** **= 398)**		
Yes	369	92.7
No	29	7.3
**Know an undergraduate colleague that have participated in research? (*N*** **= 398)**		
Yes	297	80.7
No	71	19.3
**Ever participated in any research activity outside your academic requirement? (*N*** **= 398)**		
Yes	120	32.5
No	249	67.5
**What was your role in that study? (*N*** **= 120)**		
Research Assistant	90	58.4
First Author	25	16.2
Co-Author	39	25.3
**Ever published any research paper in a peer-reviewed journal? (*N*** **= 120)**		
Yes	27	22.5
No	93	77.5
***If yes, how many articles? (N = 27)***		
*1 article*	*14*	*51.9*
*2 articles*	*6*	*22.2*
*3 or more articles*	*7*	*25.9*
***Have you had any first-author publication? (N = 27)***		
*Yes*	*11*	*40.7*
*No*	*16*	*59.3*
**What kind of journal was your paper(s) published?(*N*** **= 27)**		
International	21	70
Regional	2	6.7
Local	7	23.3
**Have you had any training on research proposal writing?(*N*** **= 398)**		
Yes	242	65.8
No	126	34.2
**Have you had any training on manuscript writing? (*N*** **= 398)**		
Yes	101	27.4
No	267	72.6
**Have you had any training on journal publication process? (*N*** **= 398)**		
Yes	68	18.5
No	300	81.5

### Attitudes towards undergraduate research

**Fig. 2:**
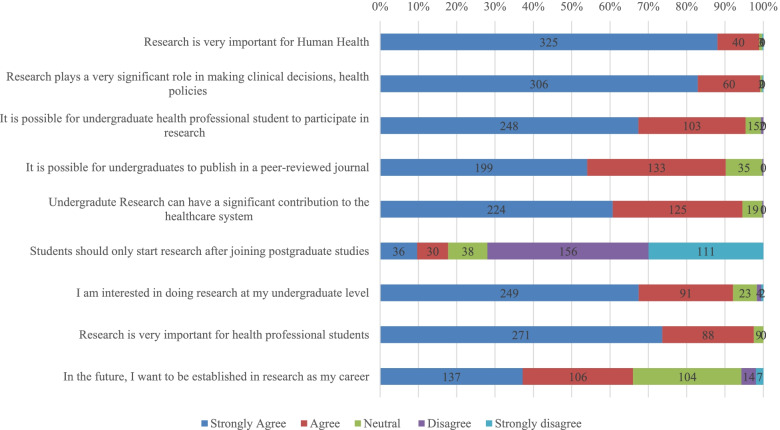
Attitude towards undergraduate research

Three hundred and twenty-five(81.6%) respondents strongly agreed that research is an important aspect in human health, and that it plays a significant role in making clinical decisions and policies (*n* = 306, 76.1%). Majority (*n* = 349, 87.6%) also believed that undergraduate research can have a significant impact on the health system of the country and 243(61.1%) were open to taking on research in their future careers (Fig. 2).

### Motivational factors and perceived barriers to participation in research

Personal development (*n* = 300, 75.4%), contribution to patient care (*n* = 294, 73.9%), gaining experience (*n* = 266, 66.8%), collaboration with senior researchers (*n* = 244, 61.3%) and developing a robust Curriculum Vita (*n* = 226, 56.8%) were the most reported motivating factors for participation in research by respondents (Table 3).

**Table 3:** Motivational factors and perceived benefits of participating in research

Question, N = 398	Frequency	Percentage
**What do you hope to benefit from conducting research?**		
Personal development	300	75.4
Acknowledgement	172	43.2
Monetary rewards	149	37.4
Contribution to patient care	294	73.9
Experience	266	66.8
Collaboration with senior researchers	244	61.3
Developing your CV by having many research papers	226	56.8
Increasing acceptability into a residency program	191	48
Passion	192	48.2
Others	4	1
**What kind of assistance do you need to improve your research participation?**		
Funding	303	76.1
Guidance on research topic selection	242	60.8
Early exposure	216	54.3
Research training	240	60.3
Providing supervisor volunteers	146	36.7
Mentorship	288	72.4
Facilitating institutional review	108	27.1
Creating a suitable environment	134	33.7
Collaboration with other researchers	184	46.2
Easing ethics approval	109	27.4
Guidance on manuscript writing	183	46
Guidance on publication of results	149	37.4
Avenues for presentation of research findings	145	36.4
Others	1	0.3
**Do you have any intentions of doing research in your future career?**		
Yes	326	81.9
No	43	10.8

Participants reported lack of funds (*n* = 317, 79.6%), lack of mentorship (*n* = 252, 63.3%), collaboration opportunities (*n* = 201, 50.5%) as the major barriers to their participation in research (Table 4). Majority of participants (*n* = 168, 42.2%) reported difficulties in study designing and manuscript writing (*n* = 155, 38.9%) as the most challenging steps in conducting a research process (Table 4).

**Table 4:** Barriers to participation in research

Question	Frequency	Percentage
**What factors would limit you from conducting research?**		
Lack of mandatory courses on research methodology	161	40.5
Lack of time for research conduction	193	48.5
Lack of funds	317	79.6
Lack of collaborations	201	50.5
Lack of interest in research	55	13.8
Lack of statistical support	135	33.9
Lack of mentorship	252	63.3
Difficulty in dealing with patients	45	11.3
Difficulty in obtaining approval for the study	155	38.9
Others	9	2.3
**What are the commonest challenges you usually find when carrying out research?**		
Lack of mentorship	223	56
Lack of motivation	141	35.4
Lack of time	166	41.7
Complexity of the research process	165	41.5
Lack of opportunities like sponsorship	279	70.1
Others	7	1.8
**What type of research do you find difficult carrying out?**		
Case Report	64	16.1
Basic science	47	11.8
Retrospective clinical study	111	27.9
Prospective clinical study	114	28.6
Clinical trial	222	55.8
Cross-sectional study	82	20.6
Review articles	66	16.6
Others	12	3
**At which of the following steps of research do you find the biggest challenge?**		
Designing a study	168	42.2
Study sampling	85	21.4
Participant Recruitment	122	30.7
Biological statistics	121	30.4
Manuscript writing	155	38.9
Paper presenting	77	19.3
Others	12	3

Likewise, participants believed that they would participate in research if funding (*n* = 303, 76%), mentorship (*n* = 288, 72.2%), research training (*n* = 240, 60.0%) were availed to them. Otherwise, majority of participants (*n* = 326, 81.9%) had intentions of doing research in future.

### Factors associated with research involvement

On bivariate analysis (Table 1), age (*p* < 0.001), sex (*p* = 0.002) and year of study (*p* < 0.001) were significantly associated with participation in research activities.

Table 1 indicates that participants 25 years or older had nearly 2-fold higher odds of taking part in research activities than younger colleagues (aOR: 1.9, 95% CI: 1.2–3.2, *p* = 0.012). Male participants had 2.1-fold higher odds of being more engaged in research than their female counterparts (aOR: 2.1, 95%CI: 1.2–3.6, *p* = 0.008). Additionally, participants in higher years had higher odds of participating in research compared to first year students with increasing odds i.e., year 3 (aOR: 3.2, 95% CI: 1.1–9.3, *p* = 0.033), year 4 (aOR: 3.3, 95% CI: 1.1–9.5, *p* = 0.028) and year 5(aOR: 11.6, 95% CI: 3.2–42.1, *p* < 0.001).

## Discussion

This study, aimed at assessing the awareness, attitude, motivation factors and barriers to research involvement among health professional students in Uganda revealed that over three-quarters of respondents were aware of medical research and with a positive attitude towards it. The major motivators for research involvement were the desire for personal development and contributing towards patient care, while lack of funds and mentorship were the main barriers for the majority.

The very high awareness (92.7%) and positive attitude towards research reported in this study could be possibly because most HPS’ schools in the country have course units on research methods incorporated in their curricular. In addition, the introduction of programs aimed at boosting undergraduate research at a few Universities such as the Health Professionals Education Partnership Initiative (HEPI) at Makerere, Busitema and Kabale Universities have strived to expose students to more research work outside the one they do for their academic requirements.

Our finding agrees with those reported by previous studies among medical and Nursing students where more than half of participants reported to be aware about research [23, 24] and had positive attitude towards it [8, 24, 25], but in contrast with that by Chellaiyan and colleagues in India [26] where less than a quarter of students had a positive attitude towards medical research. Such and more programs like research results dissemination conferences aimed at exposing students to research are encouraged to better this awareness and positive attitudes. Despite this good awareness and attitude however, only one-third (32.5%) of students had engaged in research activities outside their class research work. This could be to the fact that most HPS’ schools have tight schedules with overwhelming workload that limits time for most students to engage in co-curricular activities including research. This finding is almost like one reported in India where only 34.3% of students had engaged in research activities [26] but in contrast to one by Mubuuke et al. among Ugandan graduate radiographers where 70% had actively engaged in research activities [11]. Mentorship on how to plan and balance classwork alongside co-curricular activities during medical school could help more students to actively engage in research work.

Our findings showed that less than one-fourth (22.5%) of participants had published at least one research article, and this finding is similar to previous studies in India [26] and Sweden [10] that reported that only 15 and 17.4% students respectively had published their work in peer reviewed journals. However, this finding is Lower compared to one reported among medical practitioners in Nigeria [20] where more than one-fourth (34.3%) of participants had published at least one article in a peer reviewed journal. Nevertheless, in our study, 70 % of those who had published had done so in international journals as opposed to local or regional journals. This is possibly because more international journals waiver either partially or fully on article processing charges (APCs) for authors from low-income countries compared to regional journals. APCs have been reported in the past as one of the major factors considered by Authors from resource limited settings when choosing a journal to publish their articles from [20]. Also, the perceived increased visibility and acknowledgement to the authors in international journals compared to local and regional journals could be another trigger for this preference.

Three-fourth of respondents were motivated to involve in research for personal development and contribution to patient care. This finding is congruent with that reported in Nigeria among medical practitioners [20] but in contrast with that by Pallampathy and others among students in India who reported personal interest, facilitation of foreign exams, and peer pressure as their main motivational factors for research involvement [23]. Majority (80%) of students expressed the desire to pursue a career in research. This outcome is like those reported in South Africa [24] and England [25], where majority of students exhibited a high interest in doing research as a career. With such high interests kept to implementation, more research scientists will be anticipated in future and could lead to tremendous advancements in evidence-based medical practice, hence improved quality, and outcomes of patient care. Concerned stake holders such as medical education heads, ministry of health and other drivers of the health care system are recommended to take appropriate supportive interventions for such dreams to remain vibrant and with the motivation for better health.

Majority of respondents reported lack of funds, mentorship, and collaboration as the perceived barriers to research involvement. This could be because currently, there are generally very few research grants for undergraduate students both locally and globally, the generally suboptimal mentorship programs in most Ugandan universities, as well as the relatively bigger age gap between the famous research scientists in the country and the students. This result is congruent with that found in Malaysia that reported lack of skills, funding [27] and among Pharmacy students in Saudi Arabia who reported lack of funding, lack of encouragement [10] but in contrast with that reported among medical students in India who reported difficulty in choosing a topic, collecting data, lack of time [26] and difficulty in follow up of patients [23] as the major barriers. Improvement in early mentorship in medical schools as already suggested by participants in the study by Munabi and colleagues [28] could help in curbing such obstacles.

We also report that participants aged 25 years or older, being male, and in a higher academic year of study had higher odds of being involved in research compared to those younger than 25 years, females, and in earlier years of study, respectively. This is possibly because participants at a higher age and class of study have had more exposure to the various health disciplines including research course units, seen and interacted with senior researchers in the field, hence more chances of obtaining inspiration, mentorship, and collaboration for active research. Also, students in higher years of study have adapted to the general HPS school pressure and can easily plan well to balance their academics with co-curricular activities including research- a very time requiring activity. This finding concurs with that reported by Kyaw and colleagues in Malaysia [27] and another in Sweden [10] where students of older age and in higher years of study were more knowledgeable about research than the younger and in lower years of study. It also agrees with various studies that found a higher association between male sex and research involvement [24, 25]. However, it contrasts with that reported in Saudi Arabia where age above 25 years was associated with less involvement in research [20].

Our study has some important limitations. Firstly, we used convenience method hence only responses from respondents who could manage to answer the online questionnaire were captured, and they may not be the actual representative of all health profession students in the country. Secondly, the results are based on participants’ self-reported answers without proof confirmation by the investigators, such as one’s total number of publications and the journals used, hence liable to possibility of recall bias and telling lies. However, it is a nationwide study, covering all the 12 medical schools and their respective programs of study in the country, with significant representation from each medical school, hence these results can be generalized.

## Conclusion

Despite the massive awareness for and good attitude towards research among the respondents, active research involvement and publication is still very low. Lack of funding and mentorship are the perceived barriers to research involvement. Future investments in small grant acquisition, research training and mentorship programs are recommended.

## Data Availability

The datasets used and/or analyzed during the current study are available from the corresponding author on reasonable request.

## Abbreviations

APCs: Article Processing Charges
COVID-19: Coronavirus disease-2019
HEPI: Health Professional Education Partnership Initiative
HPS: Health Profession Student
MAKCHS: Makerere University, College of Health Sciences
MBChB: Bachelor of Medicine and Bachelor of Surgery
MHREC: Mulago Hospital Research and Ethics Committee
SSA: Sub-Saharan Africa

## References

[1] Al-Shalawy FAN, Haleem A (2015). Knowledge, attitudes and perceived barriers towards scientific research among undergraduate health sciences students in the central province of Saudi Arabia. Educ Med.

[2] Mahmood Shah SM, Sohail M, Ahmad KM, Imtiaz F, Iftikhar S (2017). Grooming future physician-scientists: evaluating the impact of research motivations, practices, and perceived barriers towards the uptake of an academic career among medical students. Cureus..

[3] Ommering BWC, Wijnen-Meijer M, Dolmans DHJM, Dekker FW, van Blankenstein FM (2020). Promoting positive perceptions of and motivation for research among undergraduate medical students to stimulate future research involvement: a grounded theory study. BMC Med Educ..

[4] Milewicz DM, Lorenz RG, Dermody TS, Brass LF, National Association of MD-PhD Programs Executive Committee (2015). Rescuing the physician-scientist workforce: the time for action is now. J Clin Invest.

[5] Ommering BWC, van Blankenstein FM, Wijnen-Meijer M, van Diepen M, Dekker FW (2019). Fostering the physician-scientist workforce: a prospective cohort study to investigate the effect of undergraduate medical students’ motivation for research on actual research involvement. BMJ Open.

[6] Rashid KA, Gomathy S, Manan A (2012). The involvement of doctors in research activities of in two major hospitals in Penang, Malsaysia. MJPHM.

[7] Imafuku R, Saiki T, Kawakami C, Suzuki Y (2015). How do students’ perceptions of research and approaches to learning change in undergraduate research?. Int J Med Educ.

[8] Ryan EJ (2016). Undergraduate nursing students’ attitudes and use of research and evidence-based practice - an integrative literature review. J Clin Nurs.

[9] Bhagavathula AS, Bandari DK, Tefera YG, Jamshed SQ, Elnour AA, Shehab A (2017). The attitude of medical and pharmacy students towards research activities: a multicenter approach. Pharmacy (Basel).

[10] Alhomoud FK, AlGhalawin L, AlGofari G, AlDjani W, Ameer A, Alhomoud F (2019). Career Choices and Preferences of Saudi Pharmacy Undergraduates: A Cross Sectional Study. Saudi Pharm J.

[11] Mubuuke AG, Businge F (2019). Self-reported competence and impact of research training among medical radiography graduates from a developing country. J Med Imaging Radiat Sci.

[12] Goldhamer ME, Cohen AP, Bates DW, Cook EF, Davis RB, Singer DE, Simon SR (2009). Protecting an endangered species: training physicians to conduct clinical research. Acad Med.

[13] Weaver AN, McCaw TR, Fifolt M, Hites L, Lorenz RG (2017). Impact of elective versus required medical school research experiences on career outcomes. J Investig Med.

[14] Möller R, Shoshan M (2017). Medical students’ research productivity and career preferences; a 2-year prospective follow-up study. BMC Med Educ..

[15] Frenk J, Chen L, Bhutta ZA, Cohen J, Crisp N, Evans T, Fineberg H, Garcia P, Ke Y, Kelley P, Kistnasamy B, Meleis A, Naylor D, Pablos-Mendez A, Reddy S, Scrimshaw S, Sepulveda J, Serwadda D, Zurayk H (2010). Health professionals for a new century: transforming education to strengthen health systems in an interdependent world. Lancet..

[16] Ngeh EN (2019). Research among undergraduate biomedical students in Cameroon: contextual barriers, room for improvement. Pan Afr Med J.

[17] Houlden RL, Raja JB, Collier CP, Clark AF, Waugh JM (2004). Medical students’ perceptions of an undergraduate research elective. Med Teach.

[18] Griffin MF, Hindocha S (2011). Publication practices of medical students at British medical schools: experience, attitudes and barriers to publish. Med Teach.

[19] Langer A, Díaz-Olavarrieta C, Berdichevsky K, Villar J (2004). Why is research from developing countries underrepresented in international health literature, and what can be done about it?. Bull World Health Organ.

[20] Adefuye AO, Adeola HA, Bezuidenhout J (2018). The physician-scientists: rare species in Africa. Pan Afr Med J..

[21] “List of medical schools in Uganda – Wikipedia” https://en.m.wikipedia.org/wiki/List_of_medical_schools_in_Uganda

[22] Munabi IG, Katabira ET, Konde-Lule J. Early undergraduate research experience at Makerere University Faculty of Medicine: a tool for promoting medical research. Afr Health Sci. 2006;6(3):182–6. 10.5555/afhs.2006.6.3.182. 10.5555/afhs.2006.6.3.182 PMC1831889 17140343

[23] Pascal Iloh GU, Amadi AN, Iro OK, Agboola SM, Aguocha GU, Chukwuonye ME (2020). Attitude, practice orientation, benefits and barriers towards health research and publications among medical practitioners in Abia state, Nigeria: a cross-sectional study. Niger J Clin Pract.

[24] Meraj L, Gul N, Zubaidazain AI, Iram F, Khan AS (2016). Perceptions and attitudes towards research amongst medical students at Shifa College of medicine. J Pak Med Assoc.

[25] Alsaleem SA, Alkhairi MAY, Alzahrani MAA, Alwadai MI, Alqahtani SSA, Alaseri YFY, Alqarni MAM, Assiri SA, Alsaleem MA, Mahmood SE (2021). Challenges and barriers toward medical research among medical and dental students at King Khalid University, Abha, Kingdom of Saudi Arabia. Front Public Health.

[26] Sayedalamin Z, Halawa TF, Baig M, Almutairi O, Allam H, Jameel T, Gazzaz ZJ, Atta H (2018). Undergraduate medical research in the Gulf cooperation council (GCC) countries: a descriptive study of the students’ perspective. BMC Res Notes.

[27] Chellaiyan VG, Manoharan A, Jasmine M, Liaquathali F (2019). Medical research: perception and barriers to its practice among medical school students of Chennai. J Educ Health Promot.

[28] Pallamparthy S, Basavareddy A (2019). Knowledge, attitude, practice, and barriers toward research among medical students: A cross-sectional questionnaire-based survey. Perspect Clin Res.

[29] Bovijn J, Kajee N, Esterhuizen TM, Van Schalkwyk SC (2017). Research involvement among undergraduate health sciences students: a cross-sectional study. BMC Med Educ.

[30] Burgoyne LN, O'Flynn S, Boylan GB. Undergraduate medical research: the student perspective. Med Educ Online. 2010;15. 10.3402/meo.v15i0.5212. 10.3402/meo.v15i0.5212 PMC2939395 20844608

[31] Kyaw Soe HH, Than NN, Lwin H, Nu Htay MNN, Phyu KL, Abas AL (2018). Knowledge, attitudes, and barriers toward research: the perspectives of undergraduate medical and dental students. J Educ Health Promot.

[32] Munabi IG, Buwembo W, Joseph R, Peter K, Bajunirwe F, Mwaka ES (2016). Students’ perspectives of undergraduate research methods education at three public medical schools in Uganda. Pan Afr Med J..

